# Preoperative nutritional screening by the specialist instead of the nutritional risk score might prevent excess nutrition: a multivariate analysis of nutritional risk factors

**DOI:** 10.1186/s12937-015-0024-1

**Published:** 2015-04-16

**Authors:** Fabian Grass, Martin Hübner, Markus Schäfer, Pierluigi Ballabeni, Yannick Cerantola, Nicolas Demartines, François P Pralong, Pauline Coti Bertrand

**Affiliations:** 1 Department of Visceral Surgery, University Hospital CHUV, Bugnon 46, 1011 Lausanne, Switzerland; 2 Institute for Social and Preventive Medicine, University Hospital CHUV, Lausanne, Switzerland; 3 Department of Urology, University Hospital CHUV, Lausanne, Switzerland; 4 Clinical Nutrition Unit, University Hospital CHUV, Lausanne, Switzerland

**Keywords:** Nutrition, Screening, Malnutrition, Perioperative, Abdominal surgery, Activity, Complications

## Abstract

**Background:**

The aim of the current study was to assess whether widely used nutritional parameters are correlated with the nutritional risk score (NRS-2002) to identify postoperative morbidity and to evaluate the role of nutritionists in nutritional assessment.

**Methods:**

A randomized trial on preoperative nutritional interventions (NCT00512213) provided the study cohort of 152 patients at nutritional risk (NRS-2002 ≥3) with a comprehensive phenotyping including diverse nutritional parameters (n=17), elaborated by nutritional specialists, and potential demographic and surgical (n=5) confounders. Risk factors for overall, severe (Dindo-Clavien 3-5) and infectious complications were identified by univariate analysis; parameters with *P*<0.20 were then entered in a multiple logistic regression model.

**Results:**

Final analysis included 140 patients with complete datasets. Of these, 61 patients (43.6%) were overweight, and 72 patients (51.4%) experienced at least one complication of any degree of severity. Univariate analysis identified a correlation between few (≤3) active co-morbidities (OR=4.94; 95% CI: 1.47-16.56, p=0.01) and *overall* complications. Patients screened as being malnourished by nutritional specialists presented *less* overall complications compared to the not malnourished (OR=0.47; 95% CI: 0.22-0.97, p=0.043). *Severe* postoperative complications occurred more often in patients with low lean body mass (OR=1.06; 95% CI: 1-1.12, p=0.028). Few (≤3) active co-morbidities (OR=8.8; 95% CI: 1.12-68.99, p=0.008) were related with postoperative *infections.* Patients screened as being malnourished by nutritional specialists presented *less* infectious complications (OR=0.28; 95% CI: 0.1-0.78), p=0.014) as compared to the not malnourished.

Multivariate analysis identified few co-morbidities (OR=6.33; 95% CI: 1.75-22.84, p=0.005), low weight loss (OR=1.08; 95% CI: 1.02-1.14, p=0.006) and low hemoglobin concentration (OR=2.84; 95% CI: 1.22-6.59, p=0.021) as independent risk factors for *overall* postoperative complications. Compliance with nutritional supplements (OR=0.37; 95% CI: 0.14-0.97, p=0.041) and supplementation of malnourished patients as assessed by nutritional specialists (OR=0.24; 95% CI: 0.08-0.69, p=0.009) were independently associated with decreased *infectious* complications.

**Conclusions:**

Nutritional support based upon NRS-2002 screening might result in overnutrition, with potentially deleterious clinical consequences. We emphasize the importance of detailed assessment of the nutritional status by a dedicated specialist before deciding on early nutritional intervention for patients with an initial NRS-2002 score of ≥3.

## Background

The selection of surgical patients at nutritional risk is mandatory since early detection and treatment of malnutrition contribute to decrease postoperative morbidity after major gastrointestinal (GI) surgery [1,2]. Currently, a number of validated and easy to use screening tools are available [2-5]. However, recently published surveys demonstrate that evidence-based guidelines for screening for malnutrition and for shaping nutritional interventions are rarely implemented outside centers with a special interest in clinical nutrition [6,7]. Instead, various clinical and laboratory parameters are preferred. A survey among Austrian and Swiss hospitals demonstrated that loss of weight, together with body mass index, were the clinical parameters most commonly used in this setting, whereas serum albumin and pre-albumin levels were the preferred laboratory parameters [6]. In that study, the screening tool currently recommended by the European Society for Parenteral and Enteral Nutrition, the Nutritional Risk Score (NRS-2002), was used by 14% of centers only [6].

The aims of the current study were two-fold: First, to assess whether the large spectrum of clinical, biological and anthropometric items correlated with the NRS-2002 to identify postoperative morbidity; and secondly, to evaluate the particular role of nutritional specialists in preoperative nutritional assessment. To achieve these goals, we used data collected during a recently published randomized trial on preoperative nutrition in patients undergoing major gastro-intestinal (GI) surgery [8]. Since all patients underwent a comprehensive nutritional assessment, we took this unique opportunity to correlate preoperative nutritional parameters with patient outcome in subjects who were all identified as high-risk patients by means of the NRS-2002 and hence receiving oral nutritional supplements.

## Methods

### Patients

The study cohort of 152 patients was provided by a recently performed double-blinded randomized study on preoperative nutrition in patients undergoing elective major GI surgery (NCT00512213) in a University Institution in Western Switzerland [8]. Major GI surgery was defined as any esophageal, gastric, hepatic, pancreatic, intestinal, and colorectal resection for benign or malignant disease and including other intra-abdominal open or laparoscopic procedures lasting more than two hours. Demographic information included age, gender, Charlson co-morbidity index [9] and the underlying pathology classified in upper GI, lower GI and retroperitoneal or hepatopancreatobiliary (HPB) disease.

All patients were routinely screened by means of the NRS-2002 [2,10], which integrates the nutritional status of the patient, the severity of the disease or planned intervention and patients’ age in a multimodal screening system. This score has been prospectively validated to identify patients who should benefit from a nutritional intervention. Patients having a NRS-2002 score of ≥3 were considered at risk for malnutrition and hence, eligible for the randomized controlled trial which provided the study cohort [8]. All patients received preoperative nutritional support, either immunonutrition® (IN), or isocaloric, isonitrogenous nutritional (ICN) supplements (6). To document compliance, patients were instructed to report effective oral intake of the allocated nutritional regimen day by day in a dedicated diary. Patients tolerating at least two thirds of the recommended dose were considered compliant, which is in line with current recommendations [11]. Patients were advised to take supplements right after the main meal in order to not reduce appetite.

### Preoperative evaluation of nutritional risk and nutrition-related parameters

Preoperative nutritional assessment was performed seven to ten days before surgery by hospital nutritionists in an outpatient setting. Body weight was measured, preoperative BMI (cutoff: 25 kg/m^2^) and weight loss were calculated, and the time span was recorded. Mid upper-arm muscle circumference was assessed by measuring the tricipital skin fold with an Adipometer Skinfold Caliper^©^, and mid upper-arm circumference was measured using a standard measuring tape. Lean body mass was assessed by bioelectrical impedance analysis (BIA-101, RJL Systems©, Akern, Italy). Further, overall physical activity level was assessed according to the lifestyle, and stratified from inactive (score 1) to extremely active (score 5) [12]. To monitor daily energy and protein intake, patients were instructed to prospectively self-assess intake by means of a dedicated diary. Patients were assisted in filling in the diary by dedicated nurses and nutritionists. Energy and protein intake were evaluated from a 24 h dietary recall. Accuracy of the reported data was cross-checked by random samples to minimize under- and overreporting. Energy and protein needs were calculated according to ESPEN guidelines, using the actual or usual body weight: 25–35 kcal/kg/day, and 0.8-1.2 g protein/kg/day, respectively [5]. Then, energy and protein gaps were calculated by comparing effective energy and protein intake with energy and protein needs. Patients were asked about the presence of dysgeusia or nausea and about abnormal gastrointestinal transit, regrouped as either diarrhea or constipation. Finally, blood samples were obtained for measurement of pertinent nutrition-related serum levels: pre-albumin <0.2 g/l (normal range 0.2-3.6 g/l), albumin <35 g/l (normal range 35–55 g/l) hemoglobin <133 g/l in men (normal range 133–172 g/l in men) or <117 g/l in women (normal range 117–156 g/l in women), and a C-reactive protein <10 mg/l (normal value <10 mg/l).

For the purpose of this present study, 17 preoperative nutritional parameters and 5 potential demographic and surgical confounders were extracted from the initial database (Table 1). Hospitals’ nutritional specialists performed nutritional assessment according to weight loss and anthropometric measures and stratified patients as being not malnourished (AM = absent malnutrition), moderately (MM) or severely malnourished (SM) (Table 2). All these parameters were then correlated with postoperative morbidity and compared with the NRS-2002 as a risk-identifying score.

**Table 1:** **Univariate risk factors for overall complications**

Item	Patients with complication (n = 72)	Patients without complication (n = 68)	Overall (n = 140)	OR (95% CI)
***Demographics:***				
**Age** (mean ± SD)	69 ± 14	66 ± 14	68 ± 14	1.01 (0.99-1.04)
**>70 yrs**	40 (56%)	38 (56%)	78 (56%)	0.99 (0.51-1.92)
**Gender** (M : F)	45 : 27	33 : 35	78 : 62	1.77 (0.9-3.45)
**Type of surgery:**				
upper GI	16 (22%)	8 (12%)	24 (17%)	2.54 (0.94-6.85)^1^
lower GI/retroperitoneal	30 (42%)	27 (40%)	57 (41%)	1.41 (0.68-2.93)^1^
HPB	26 (36%)	33 (48%)	59 (42%)	1
**Nutritional support type:**				
IN : ICN	38 : 34	31 : 37	69 : 71	1.33 (0.69-2.59)
**Charlson comorbidity index:**				
0	4 (6%)	12 (18.5%)	16 (12%)	1
1-3	56 (78%)	34 (52%)	90 (66%)	**4.94 (1.47-16.56)** ^2^
4-7	6 (8%)	12 (18.5%)	18 (13%)	1.5 (0.34-6.7)^2^
>7	6 (8%)	7 (11%)	13 (9%)	2.57 (0.53-12.38)
>3	25 (35%)	24 (35%)	49 (36%)	1.15 (0.75-1.74)
***Nutritional parameters:***				
***Clinical***				
**NRS-2002** (mean ± SD)	3.7 ± 1.0	4.0 ± 1.0	3.8 ± 1.0	0.77 (0.54-1.09)
**Nutritional diagnosis:**				
AM	35 (49%)	22 (32%)	57 (41%)	1
MM	26 (36%)	35 (52%)	61 (44%)	**0.47 (0.22-0.97)** ^3^
SM	11 (15%)	11 (16%)	22 (16%)	0.63 (0.23-1.69)^3^
**Weight difference/initial**	−5.9 ± 8.7	−8.4 ± 7.1	−7.1 ± 8.1	1.04 (0.99-1.09)
**weight (%)** (mean ± SD)				
**Weight loss >10%**	21 (29%)	22 (32%)	43 (31%)	0.86 (0.42-1.77)
**Time span of weight loss (d)** (mean ± SD)	138 ± 167	170 ± 251	154 ± 212	1 (0.99-1.01)
**BMI (kg/m**^**2**^**)** (mean ± SD)	23.7 ± 4.2	23.1 ± 4.1	23.4 ± 4.2	1.03 (0.96-1.13)
**BMI (kg/m** ^**2**^ **) > 25**	35 (49%)	26 (38%)	61 (44%)	1.53 (0.78-3)
**Lean body mass (kg)** (mean ± SD)	48.3 ± 9.0	46.5 ± 9.5	47.4 ± 9.2	1.02 (0.98-1.06)
**% lean body mass** (mean ± SD)	70.7 ± 10.2	71.9 ± 9.3	71.3 ± 9.8	0.99 (0.95-1.03)
**Mid upper-arm muscle circumference** (cm) (mean ± SD)	244.1 ± 27.9	237.5 ± 33.2	240.9 ± 31	1.01 (0.99-1.02)
**Physical activity score 3+**	20 (28%)	21 (31%)	41 (29%)	0.86 (0.42-1.78)
**Energy gap (kcal)** (mean ± SD)	−270 ± 548	−326 ± 692	−297 ± 620	1 (0.99-1)
**Protein gap (g)** (mean ± SD)	−2.4 ± 23.2	−2.9 ± 24.7	−2.6 ± 23.9	1 (0.99-1.01)
**Compliance with allocated nutritional support** (>10/15)	40 (56%)	41 (60%)	81 (58%)	0.82 (0.42-1.61)
***Biological***				
**Albumin** (<35 g/l)	8 (11%)	5 (7%)	13 (9%)	1.6 (0.49-5.17)
**Prealbumin** (<0.2 g/l)	23 (32%)	24 (35%)	47 (34%)	0.87 (0.43-1.78)
**CRP** (<10 mg/l)	25 (35%)	19 (28%)	44 (31%)	1.38 (0.66-2.88)
**Hemoglobin**	41 (57%)	28 (41%)	69 (49%)	1.88 (0.95-3.75)
(<133 g/l for men;				
<117 g/l for women)				

Results from logistic regressions. An OR > 1 means an increased likelihood of complications.

HPB – hepatopancreatobiliary.

IN – immunonutrition, ICN – isocaloric-isonitrogenous nutrition.

BMI ─ Body Mass Index.

NRS-2002 ─ Nutritonal Risk Score.

AM ─ Absent Malnutrition, MM ─ Moderate Malnutrition, SM ─ Severe Malnutrition.

Bold numbers indicate p < 0.05.

^1^OR is calculated with hepatopancreatobiliary surgery as a reference.

^2^OR is calculated with Charlson score = 0–1 as a reference.

^3^OR is calculated with AM as a reference. The global test for all nutritional categories is statistically insignificant (p = 0.127).

**Table 2:** **Criteria used to assess the level of preoperative malnutrition**

	Weight loss during the past 6 months in %	MAMC	FFM
**Absence of malnutrition**	<5%	-	-
**Moderate malnutrition**	5 - 19%	>5^th^ percentile	>5^th^ percentile
**Severe malnutrition**	≥20%	≤5^th^ percentile	≤5^th^ percentile

MAMC: mid-arm muscle circumference.

FFM: fat-free-mass measured by bio-impedancemetry.

As indicated in the table, there are different criteria for severe and moderate malnutrition depending on weight loss and body mass composition.

### Outcomes/study endpoints

The primary endpoint for the present analysis was overall complication rate. Postoperative complications (30-day morbidity) were graded according to their severity on a validated therapy-orientated scale [13]. Complications were reported as number of complications. Hence, more than one complication per patient was possible. Secondary endpoints were infectious and severe complications. The latter were defined as complications grade 3–5 according to the Dindo-Clavien classification [13]. All postoperative infections were accounted for as infectious complications, including wound infections, intra-abdominal abscesses, pneumonia, urinary tract infections and sepsis.

### Data synthesis and analysis

Descriptive statistics are reported as median (range) or mean (±SD) for continuous variables and absolute or relative frequencies for categorical variables. Logistic regressions were used to test the effect of nutritional variables on the binary outcomes overall complications, severe complications and infectious complications. Each outcome was analyzed separately. First, dependent variables were tested individually in simple regressions. Variables with *P*-values ≤ 0.2 were entered into a multiple logistic regression to provide adjusted estimations of the odds-ratio (OR). All tests were 2-tailed. A *P*-value of less than 0.05 was considered statistically significant.

Data analysis was performed with Prism 5.2 (GraphPad® Software, Inc. 2236 Avenida de la Playa La Jolla, CA 92037 USA) and Stata, version 11.0 (StataCorp LP, College Station, TX).

## Results

For the purpose of the present study, final analysis included 140 patients with complete datasets. Of these, 61 patients (43.6%) were overweight or obese, and 72 patients (51.4%) experienced at least one complication of any degree of severity. One hundred and nineteen (85%) patients suffered of malignant disease. Of these, 45 patients (38%) needed a systemic oncological approach with neoadjuvant therapy before surgical management. We observed caloric and protein depletion with a respective gap of 300 (±600) kcal and 3 (±24) grams, respectively, with no significant differences in patients with and without complications. Forty-three patients (31%) presented with weight loss of > 10%. Twenty-six patients (19%) lost > 10% of their body weight in less than 6 months. Thirty-six patients (26%) lost > 5% in less than 4 weeks.

### Risk factors for overall complications (Clavien grade 1–5)

A patient-related univariate risk factor for increased *overall* complication rates was the presence of few (≤ 3) active co-morbidities (OR = 4.94; 95% CI: 1.47-16.56, p = 0.01). We observed several trends: Male gender (OR = 1.77; 95% CI: 0.9-3.45, p = 0.097) and upper GI surgery as compared to hepatopancreatobiliary surgery (OR = 2.54; 95% CI: 0.94-6.85, p = 0.065) correlated with postoperative complications. Surprisingly, patients without complications had more weight loss preoperatively than those without complications (OR = 1.04; 95% CI: 0.99-1.09, p = 0.063) (Table 1). Further, patients screened as being moderately malnourished by nutritional specialists presented *less* overall complications compared to those who were screened as not malnourished (OR = 0.47; 95% CI: 0.22-0.97, p = 0.043). We observed higher overall complication rates in patients with abnormal hemoglobin levels (OR = 1.88; 95% CI: 0.95-3.75, p = 0.072).

After multivariate analysis, the presence of few active comorbidities (OR = 6.33; 95% CI: 1.75-22.84, p = 0.005), low recent weight loss (OR = 1.08; 95% CI: 1.02-1.14, p = 0.006) and low hemoglobin concentration (OR = 2.84; 95% CI: 1.22-6.59, p = 0.021) were retained as independent risk factors for increased postoperative *overall* complications.

### Risk factors for severe complications (Clavien grade 3–5)

*Severe* complications were observed in 29 patients (20.7%). Patient-related univariate risk factors for increased *severe* complication rates was low lean body mass (OR = 1.06; 95% CI: 1–1.12, p = 0.028) and a trend towards increased risk was identified for low body mass index (OR = 1.09; 95% CI: 0.99-1.21, p = 0.082) and low albumin concentration (OR = 2.78; 95% CI: 0.83-9.33, p = 0.097). Multivariate analysis identified no independent risk factor for increased postoperative *severe* complications.

### Risk factors for infectious complications

At least one infectious complication was observed in 26 patients (18.6%).

A patient-related univariate risk factor for increased *infectious* complication rates was the presence of few (≤3) active co-morbidities (OR = 8.8; 95% CI: 1.12-68.99, p = 0.008). The group of patients with moderate malnutrition presented *less* infectious complications (OR = 0.28; 95% CI: 0.1-0.78), p = 0.014) as compared to the not-malnourished group after univariate analysis. Further, a trend was observed towards *less* infectious complications in the compliant patient group (OR = 0.46; 95% CI: 0.19-1.09, p = 0.079). Retained as independent risk factor for infectious complications after multivariate analysis were few active co-morbidites (OR = 10.6; 95% CI: 1.3-86.23, p = 0.004). Independent protective factors for infectious complications were high compliance with the respective nutritional intervention (OR = 0.37; 95% CI: 0.14-0.97, p = 0.041) and moderate malnutrition (OR = 0.24; 95% CI: 0.08-0.69, p = 0.009) as compared to absent malnutrition.

Results of uni- and multivariate analysis are illustrated in Tables 3 and 4.

**Table 3:** **Univariate analysis of any, severe and infectious complications with P < 0.1**

Item	Patients with any complication (n = 72/140)	Patients with severe complication (n = 29/140)	Patients with infectious complication (n = 26/140)
	**OR (95% CI)**	**OR (95% CI)**	**OR (95% CI)**
**Gender** (M : F)	1.77 (0.9-3.47)	2.03 (0.85-4.85)	2.02 (0.81-5.03)
**Type of surgery:**			
upperGI	2.54 (0.94-6.85)^1^	1.45 (0.47-4.51)^1^	1.8 (0.6-5.38)^1^
lowerGI/retroperitoneal	1.41 (0.68-2.93)^1^	1.16 (0.47-2.9)^1^	0.71 (0.26-1.92)^1^
HPB	1	1	1
**Charlson index:**			
0	1	1	1
1-3	**4.94 (1.47-16.56)** ^**2**^	2.13 (0.45-10.14)^2^	**8.8 (1.12-68.99)** ^**2**^
4-7	1.5 (0.33-6.7)^2^	1.4 (0.2-9.66)^2^	2.67 (0.26-27.38)^2^
>7	2.57 (0.53-12.38)^2^	2.01 (0.94-14.98)^2^	1.84 (0.34-16.34)^2^
**Nutritional diagnosis:**			
AM	1	1	1
MM	**0.47 (0.22-0.97)** ^**3**^	0.55 (0.22-1.35)^3^	**0.28 (0.1-0.78)** ^**3**^
SM	0.62 (0.23-1.69)^3^	0.62 (0.18-2.14)^3^	0.57 (0.17-1.94)^3^
**BMI**	1.04 (0.96-1.13)	1.09 (0.99-1.21)	1.02 (0.92-1.13)
**Lean body mass**	1.02 (0.98-1.06)	**1.06 (1.01-1.12)**	1 (0.95-1.05)
**Compliance with allocated nutritional support** (>10/15)	0.82 (0.42-1.61)	1.5 (0.64-3.52)	0.46 (0.19-1.09)
**Albumin** (<35 g/l)	1.60 (0.5-5.17)	2.78 (0.83-9.33)	0.81 (0.17-3.92)
**Hemoglobin** (<133 g/l for men; <117 g/l for women)	1.88 (0.95-3.75)	1.31 (0.56-3.03)	1.25 (0.51-3.1)

Results from logistic regressions. An OR > 1 means an increased likelihood of complications.

HPB ─ hepatopancreatobiliary.

BMI ─ Body Mass Index.

AM ─ Absent Malnutrition, MM ─ Moderate Malnutrition, SM ─ Severe Malnutrition.

OR (95% CI) ─ Odds ratio (95% Confidence Interval).

Bold numbers indicate p < 0.05.

^1^P is calculated with hepatopancreatobiliary surgery as a reference.

^2^P is calculated with Charlson score = 0–1 as a reference.

^3^P is calculated with AM as a reference.

**Table 4:** **Multivariate analysis of any, severe and infectious complications**

Item	Patients with any complication (n = 72/140)	Patients with severe complication (n = 29/140)	Patients with infectious complication (n = 26/140)
	OR (95% CI)	OR (95% CI)	OR (95% CI)
**Gender** (M : F)	2.04 (0.93-4.5)	*NR*	*NR*
**Charlson index:**			
0	1	*NR*	1
1–3	**6.33 (1.75-22.84)** ^**1**^		**10.6 (1.3-86.23)** ^**1**^
3–7	1.47 (0.3-7.13)^1^		3.45 (0.32-37.18)^1^
>7	3.1 (0.56-17.16)^1^		3.24 (0.47-16.28)^1^
**Nutritional diagnosis:**			
AM	*NR*	*NR*	1
MM			**0.24 (0.08-0.69)** ^**2**^
SM			0.44 (0.12-1.68)^2^
**Weight difference/initial weight** (%)	**1.08 (1.02-1.14)**	1.06 (0.99-1.14)	*NR*
**BMI**	*NR*	0.99 (0.86-1.14)	*NR*
**Lean body mass**	*NR*	1.06 (1–1.13)	*NR*
**Compliance with allocated nutritional support** (>10/15)	*NR*	*NR*	**0.37 (0.14-0.97)**
**Albumin** (<35 g/l)	*NR*	2.72 (0.63-11.76)	*NR*
**Hemoglobin** (<133 g/l for men; <117 g/l for women)	**2.84 (1.22-6.59)**	*NR*	*NR*

An OR > 1 means an increased likelihood of complications.

*NR* ─ not retained for multivariate analysis.

BMI ─ Body Mass Index.

AM ─ Absent Malnutrition, MM ─ Moderate Malnutrition, SM ─ Severe Malnutrition.

Bold numbers indicate p < 0.05.

^1^P is calculated with Charlson score = 0–1 as a reference.

^2^P is calculated with AM as a reference.

Of note, several seemingly obvious risk factors for postoperative adverse outcomes did not show any significant statistical correlation with postoperative morbidity. These were in particular serum biochemistry values such as albumin, prealbumin or CRP, anthropometric measures such as the mid upper-arm muscle circumference and assessment of energy and protein gaps.

## Discussion

Identifying patients at risk of developing postoperative complications remains a challenging clinical objective. The nutritional status of any given individual is generally considered as an important component of this risk, and a number of nutritional indicators have been proposed, ranging from easily assessable parameters, such as weight loss of more than 10% in 6 months or decreased recent food intake [14], to biochemical markers or physiologic and anthropometric measurements [15-21]. In this study, we took advantage of data generated during a previously published study (6) to assess the potential usefulness in predicting postoperative morbidity of a large number of clinical and biochemical parameters obtained by a comprehensive nutritional phenotyping. Surprisingly, in this analysis, no single nutritional parameter was a strong predictor for postoperative morbidity on its own, partly in contrast to available evidence [22,23]. Only anemia has been retained as independent risk factor for overall complications among the nutrition-related factors. Hemoglobin is part of most routinely performed preoperative blood samples before major surgery [21]. Anemia correlates with cancer related hematochezia and reflects poor nutritional status [24]. Lean body mass has been associated with prolonged hospital stay and in-hospital morbidity when decreased [17]. In the present study, the rate of severe complications was slightly higher in patients with low lean body mass.

One recommended screening tool by the ESPEN society is the NRS-2002 which integrates patients’ age, the magnitude of the intervention and patient’s nutritional status in a multimodal screening tool [2,10]. This score was applied to select the patient cohort for the randomized controlled trial and thus for the study cohort of the present analysis, based on a score of 3 at least. A NRS-2002 score of 3 can be achieved by advanced age alone (1 point), since every patient already scored 2 points for undergoing major surgery [2]. Further, it depicts only the actual situation, and it may vary very rapidly. Hence, these elderly patients did not necessarily suffer from metabolic imbalance due to recent weight loss or muscle wasting, and thus the NRS-2002 does not necessarily reflect the actual metabolic state of these patients. In this respect, it is remarkable that following nutritional assessment by the specialist, 41% of the selected patients were not considered malnourished, suggesting that the nutritional intervention should not have been systematically started preoperatively based only on the NRS-2002 score, as proposed in the original publication of Kondrup et al. [2].

The high complication rate in our series can be explained by a very meticulous prospective documentation of *all* adverse events for the purpose of the study; our results reflect what is probably the reality if outcome is monitored completely and with sufficient follow-up [25,26]. Remarkably, the overall complication rate was higher in well-nourished patients as assessed preoperatively by the nutritional specialist than in malnourished subjects. Further, patients with a low index of comorbidities had a higher rate of overall postoperative complications when compared to patients with a higher index of comorbidities. Together, these somewhat surprising results may suggest the presence, in this population, of an aggravating factor.

A potentially important difference between the present data and previously published results is the systematic application of a preoperative nutritional intervention. Indeed, all patients of the cohort, irrespective of the results of the nutritional evaluation performed by the specialist, received oral nutritional supplements. In other words, the subgroup of well-nourished patients who experienced a higher complication rate than malnourished subjects based on this evaluation, received the same additional 909 Kcal/day starting 5 days before surgery. Since our patient cohort presented with a mean caloric gap of about 300 kcal and a mean protein gap of about 3 g, and considering that physical activity was important in one third of patients only, this daily caloric intake might have led to caloric excess. This has been shown so far for the postoperative period [27]. It was also shown that a hyperglycemic state, generated by supplementation of metabolically stable patients, creates insulin resistance, which in turn complicates the maintenance of a perioperative anabolic state [28].

We therefore hypothesize that the higher complication rate observed in the well-nourished sub-group of patients may be linked to this inappropriate caloric load, in line with the findings of Gianotti et al. who demonstrated higher complication rates in well-nourished subgroups [29]. This is even more interesting in the setting of the major health problem obesity in the general population nowadays. While 36% of patients had a BMI of ≥ 25 kg/m^2^ in the cohort of Gianotti et al. in 2000 [29], we dealt with 44% of patients with a BMI of ≥ 25 kg/m2 in our cohort.

Our results also resonate with previously published data showing that nutritional intervention was beneficial only in patients with an initial NRS-2002 score of ≥ 5 [30]. In this study, a significant reduction in complication rates of 50% was reported in these patients when compared to patients in the control group. The authors conclude that an NRS-2002 exceeding 5 probably indicates that the subjects are actually malnourished, and therefore likely benefit the most of preoperative nutritional support. Our own data are consistent with this hypothesis, and further suggest that preoperative nutritional support in patients that are not malnourished might be deleterious. Of note, the finding that subjects experiencing less preoperative weight loss had a higher complication rate might further support this hypothesis. This particular subset of patients might not have been malnourished, and thus was exposed to hyper-caloric feeding by our protocol.

Several limitations of this study need to be addressed: The patient cohort for the present study has been selected based on a NRS-2002 score of 3 at least. This means that comparison with a control group of low risk patients with regard to the NRS-2002 score (< 3) was not possible. The assessment has been performed in a highly motivated center, however with a limited experience in perioperative nutritional screening and therapy. A majority of patients (85%) suffered of malignant disease, and about one third of them needed neoadjuvant therapy before surgical management, which might possibly bias some of the analyzed parameters.

## Conclusions

Our data suggest that early nutritional support based upon NRS-2002 only might result in overnutrition, with potentially deleterious clinical consequences post-operatively. In line with the recommendations of Kondrup et al. [2], we emphasize the importance of detailed assessment of the nutritional status by a dedicated specialist before deciding on early nutritional intervention in patients with an initial NRS-2002 score of 3 or more. In this respect, the NRS-2002 score should not be misconceived as easy tool to replace proper nutritional assessment. Those patients who do not qualify for early nutritional support should be followed closely during the perioperative period, in order to ascertain the optimal time point at which nutritional support should be introduced. However, this strategy should be formally evaluated by properly conducted randomized and controlled trials.

## Abbreviations

GI: Gastrointestinal
NRS-2002: Nutritional risk score
ONS: Oral nutritional supplement

## References

[1] Weimann A, Breitenstein S, Breuer JP, Gabor SE, Holland-Cunz S, Kemen M (2014). [Clinical nutrition in surgery : Guidelines of the German Society for Nutritional Medicine]. Chirurg.

[2] Kondrup J, Allison SP, Elia M, Vellas B, Plauth M (2003). ESPEN guidelines for nutrition screening 2002. Clin Nutr.

[3] Cerantola Y, Grass F, Cristaudi A, Demartines N, Schafer M, Hubner M (2011). Perioperative nutrition in abdominal surgery: recommendations and reality. Gastroenterol Res Pract.

[4] Schindler K, Pernicka E, Laviano A, Howard P, Schutz T, Bauer P (2010). How nutritional risk is assessed and managed in European hospitals: a survey of 21,007 patients findings from the 2007–2008 cross-sectional nutritionDay survey. Clin Nutr.

[5] Weimann A, Braga M, Harsanyi L, Laviano A, Ljungqvist O, Soeters P (2006). ESPEN Guidelines on Enteral Nutrition: Surgery including organ transplantation. Clin Nutr.

[6] Grass F, Cerantola Y, Schafer M, Muller S, Demartines N, Hubner M (2011). Perioperative nutrition is still a surgical orphan: results of a Swiss-Austrian survey. Eur J Clin Nutr.

[7] Agarwal E, Ferguson M, Banks M, Batterham M, Bauer J, Capra S (2012). Nutrition care practices in hospital wards: results from the Nutrition Care Day Survey 2010. Clin Nutr.

[8] Hubner M, Cerantola Y, Grass F, Bertrand PC, Schafer M, Demartines N (2012). Preoperative immunonutrition in patients at nutritional risk: results of a double-blinded randomized clinical trial. Eur J Clin Nutr.

[9] de Groot V, Beckerman H, Lankhorst GJ, Bouter LM (2003). How to measure comorbidity. a critical review of available methods. J Clin Epidemiol.

[10] Kondrup J, Rasmussen HH, Hamberg O, Stanga Z (2003). Nutritional risk screening (NRS 2002): a new method based on an analysis of controlled clinical trials. Clin Nutr.

[11] McClave SA, Martindale RG, Vanek VW, McCarthy M, Roberts P, Taylor B (2009). Guidelines for the Provision and Assessment of Nutrition Support Therapy in the Adult Critically Ill Patient: Society of Critical Care Medicine (SCCM) and American Society for Parenteral and Enteral Nutrition (A.S.P.E.N.). JPEN J Parenter Enteral Nutr.

[12] Vermorel M, Ritz P, Tappy L, Laville M, Martin A (2001). Apports nutritionnels conseillés pour la population française.

[13] Dindo D, Demartines N, Clavien PA (2004). Classification of surgical complications: a new proposal with evaluation in a cohort of 6336 patients and results of a survey. Ann Surg.

[14] Braga M, Gianotti L, Nespoli L, Radaelli G, Di Carlo V (2002). Nutritional approach in malnourished surgical patients: a prospective randomized study. Arch Surg.

[15] Seres DS (2005). Surrogate nutrition markers, malnutrition, and adequacy of nutrition support. Nutr Clin Pract.

[16] Barbosa-Silva MC, Barros AJ (2005). Bioelectric impedance and individual characteristics as prognostic factors for post-operative complications. Clin Nutr.

[17] Pichard C, Kyle UG, Morabia A, Perrier A, Vermeulen B, Unger P (2004). Nutritional assessment: lean body mass depletion at hospital admission is associated with an increased length of stay. Am J Clin Nutr.

[18] Antoun S, Rey A, Beal J, Montange F, Pressoir M, Vasson MP (2009). Nutritional risk factors in planned oncologic surgery: what clinical and biological parameters should be routinely used?. World J Surg.

[19] Ranzani OT, Zampieri FG, Forte DN, Azevedo LC, Park M (2013). C-reactive protein/albumin ratio predicts 90-day mortality of septic patients. PLoS One.

[20] Robinson MK, Trujillo EB, Mogensen KM, Rounds J, McManus K, Jacobs DO (2003). Improving nutritional screening of hospitalized patients: the role of prealbumin. JPEN J Parenter Enteral Nutr.

[21] Brugler L, Stankovic AK, Schlefer M, Bernstein L (2005). A simplified nutrition screen for hospitalized patients using readily available laboratory and patient information. Nutrition.

[22] Schiesser M, Muller S, Kirchhoff P, Breitenstein S, Schafer M, Clavien PA (2008). Assessment of a novel screening score for nutritional risk in predicting complications in gastro-intestinal surgery. Clin Nutr.

[23] Schwegler I, von Holzen A, Gutzwiller JP, Schlumpf R, Muhlebach S, Stanga Z (2010). Nutritional risk is a clinical predictor of postoperative mortality and morbidity in surgery for colorectal cancer. Br J Surg.

[24] Smith RC, Ledgard JP, Doig G, Chesher D, Smith SF (2009). An effective automated nutrition screen for hospitalized patients. Nutrition.

[25] Byrne BE, Mamidanna R, Vincent CA, Faiz O (2013). Population-based cohort study comparing 30- and 90-day institutional mortality rates after colorectal surgery. Br J Surg.

[26] Slankamenac K, Graf R, Barkun J, Puhan MA, Clavien PA (2013). The comprehensive complication index: a novel continuous scale to measure surgical morbidity. Ann Surg.

[27] Perioperative total parenteral nutrition in surgical patients. The Veterans Affairs Total Parenteral Nutrition Cooperative Study Group. N Engl J Med. 1991;325(8):525–32. 10.1056/NEJM199108223250801 1906987

[28] Evans DC, Martindale RG, Kiraly LN, Jones CM (2014). Nutrition optimization prior to surgery. Nutr Clin Pract.

[29] Gianotti L, Braga M, Nespoli L, Radaelli G, Beneduce A, Di Carlo V (2002). A randomized controlled trial of preoperative oral supplementation with a specialized diet in patients with gastrointestinal cancer. Gastroenterology.

[30] Jie B, Jiang ZM, Nolan MT, Zhu SN, Yu K, Kondrup J (2012). Impact of preoperative nutritional support on clinical outcome in abdominal surgical patients at nutritional risk. Nutrition.

